# Tailoring Fusion Proteins for Interfacial Nanoparticle Functionalization: Toward Fluorescence-Guided Imaging

**DOI:** 10.1021/acsomega.6c09485

**Published:** 2026-10-13

**Authors:** Ryu Masaki, Julia T. Lucena, Maritza A. Todo Bom, Isabela P. Dias, Mariana Vodiani, Giovanna Furman, Joslaine Jacumazo, Rilton A. de Freitas, Guinther Kellerman, Maísa Colodel, Luiz M. de L. Faria, Gabriela C. Cardoso, Edneia A. S. Ramos, Lindomar J. C. Albuquerque, Genilza da Silva Mello, Camila de Almeida Salvego, Breno C. B. Beirão, Edileusa M. Gerhardt, Emanuel Maltempi de Souza, Marcelo Müller-Santos, Guilherme F. Picheth

**Affiliations:** † Department of Biochemistry and Molecular Biology, Post-graduate Program in Biochemistry, 28122 Federal University of Paraná, Curitiba, Paraná 81531-980, Brazil; ‡ Department of Pharmacy, 28122 Federal University of Paraná, Curitiba, Paraná 80210-170, Brazil; § Department of Physics, 28122 Federal University of Paraná, Curitiba, Paraná 81531-980, Brazil; ∥ Department of Chemistry and Biology, Federal Technological University of Paraná, Curitiba, Paraná 81280-340, Brazil; ⊥ Post-Graduate Program in Microbiology, Parasitology and Pathology, Federal University of Paraná, Curitiba, Paraná 81531-980, Brazil; # Brazilian Synchrotron Light Laboratory, Brazilian Center for Research in Energy and Materials, Campinas, São Paulo 13083-100, Brazil; ∇ Araguaia University Center, Barra do Garças, Mato Grosso CEP 78.603-209, Brazil

## Abstract

Fusion proteins are specially designed molecules created by linking two or more peptides that were originally encoded by separate DNA sequences. To improve control of protein adsorption on poly­(3-hydroxybutyrate) (PHB) surfaces, we developed fusion proteins comprising a polyester substrate-binding domain (SBD) fused to superfolder green fluorescent protein (sfGFP). The subunits were connected via flexible (GGGGS)_n_ or rigid (EAAAK)_n_ linkers with n = 1, 2, or 4. The PHB immobilization efficiency provided by these fusion proteins was extensively evaluated on microparticles and nanoparticles by using various surface-sensitive techniques. Accordingly, constructs linked by rigid sequences with n = 2 and n = 4 exhibited higher PHB adsorption capability. This suggests that reduced interdomain conformational freedom may favor configurations compatible with SBD-mediated interaction with the PHB interface. The stable association of the constructs with PHB nanoparticles enabled fluorescence-based tracking during cellular uptake experiments. The spatial orientation and polyester-binding ability conferred by such constructs via a facile, mild, and persistent association process might represent a significant biotechnological advance in the development of advanced nanosystems sensitive to fluorescence imaging.

## Graphical abstract

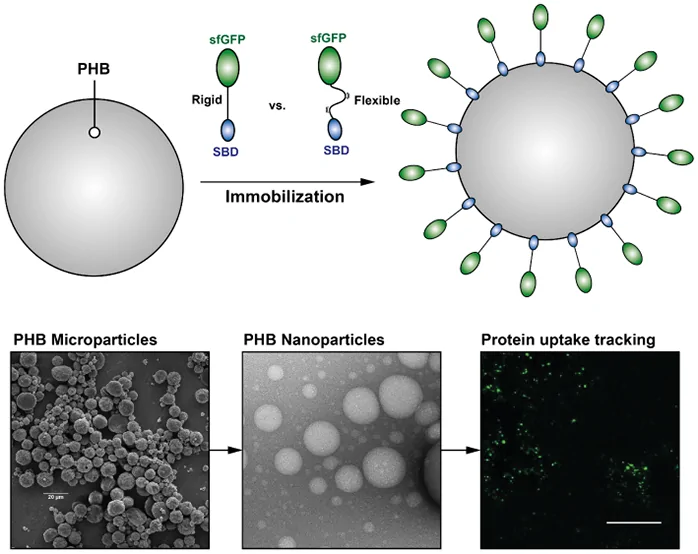

## 1 Introduction

Fusion proteins are chimeric macromolecules constituted by two or more protein sequences originally coded by different genes, all linked in a single structure.^1^ The inherent multivalent and complex structure has enabled the development of advanced therapeutic agents with precise targeting, enhanced efficacy, and improved pharmacokinetics.^2–7^ The successful translation of fusion proteins, however, is highly dependent on the 3D architecture as well as the chemical character of each domain.^8^

To provide stability and ensure optimal segmental activity, a peptide linker is inserted between the protein sequences and performs a crucial role in preserving the structural properties and interactions between the fused domains.^8,9^ In fact, several natural multidomain proteins contain interdomain linkers with varied amino acid sequences, extensions, secondary structures, and chemical characteristics.^10,11^

Fusion protein constructs are essential tools for peptide-based micro- and nanotechnology, as they may spontaneously assemble at biointerfaces.^12^ Similar systems are currently employed in a wide variety of biorelated applications, including vaccines, drug delivery systems, and diagnostic platforms.^13–17^ The performance of similar biotechnological devices relies on precise protein auto-organization along 3D surfaces, which enables the formation of homogeneous and functional interfaces. The biofunctionalization of several material-designed surfaces, however, is severely hindered due to short-range interactions between the fused domains.^18^

To provide better control of protein adsorption over poly­(3-hydroxybutyrate) (PHB) surfaces, we developed fused proteins formed by a polyester substrate-binding domain (SBD)^19,20^ linked to a fluorescent report unit, the superfolder green fluorescent protein (sfGFP)—sfGFP-SBD. Although our previous studies demonstrated that the SBD is able to mediate the immobilization of sfGFP-containing fusion proteins on PHB micro- and nanostructures, the role of the interdomain architecture in controlling this interaction still remains unexplored. In this study, we address the influence of the molecular architecture connecting the reporter and substrate-binding domains on interfacial binding to PHB. To this end, we systematically varied both linker flexibility and length using flexible (GGGGS)_n_ and rigid (EAAAK)_n_ sequences (n = 1, 2, and 4).^21^ The impact of each linker and its length on protein adsorption over PHB microparticles (MP) and nanoparticles (NP) was evaluated using various surface-sensitive techniques. The nanosystems with the best construct-immobilization performance were tracked by monitoring their fluorescence emission within cells for up to 48 h.

## 2 Methodology

### 2.1 Protein Expression and Purification

The DNA sequence encoding sfGFP was obtained from the Registry of Standard Biological Parts (parts.igem.org). Two NdeI recognition sites (CATATG) present in the original sequence were mutated while preserving the protein’s original amino acid sequence. The DNA sequence for the substrate-binding domain (SBD) from *Ralstonia pickettii* T1 PHA depolymerase was obtained from ref 22. The amino acid sequences from flexible [(GGGGS)_n_] and rigid [A­(EAAAK)_n_A] linker units were derived from ref 8. All constructs had their codons optimized for *E. coli* expression and were designed to be compatible with the pET expression system. Gene synthesis and cloning into pET29b­(+) plasmids were performed by GenScript (USA). The pET28a­(+)_sfGFP-SBD plasmid, used for expression of the original sfGFP-SBD fusion protein, was obtained from ref 23. The DNA sequences and annotated features of each construct are available in Table S1. Overexpression and protein purification processes were performed as previously reported.^20^

### 2.2 Small-Angle X-ray Scattering

The SAXS experiments were performed at the Brazilian Synchrotron Light (LNLS) facility. The instrument was previously calibrated with silver behenate. All samples were measured at room temperature, detector distance = 3 m, and sample exposure = 10 s and 30 s. Before data processing, all corrections were applied to the two-dimensional image obtained during the experiment. All samples were diluted to 0.2 mg.mL^–1^ in Tris-HCl buffer (pH 8.0) prior to analysis. SAXS data modeling was performed using SASfit software.

### 2.3 3D Protein Structure Prediction

The 3D structure of all constructs was predicted using AlphaFold3.^24^ The CIF file with the highest confidence score was further processed and exported to ChimeraX^25^ and Blender.

### 2.4 Microparticle Formulation and Characterization

PHB microparticles were produced through an emulsion-solvent evaporation approach.^26^ Briefly, 25 mg of PHB (Sigma-Aldrich) was dissolved in 2 mL of chloroform. Subsequently, 10 mL of 1% (w/v) poly­(vinyl alcohol) (PVA, *M_w_* 31 kDa, 88% hydrolyzed) solution was added to the organic phase, and the mixture was homogenized at high speed for 1 min. The emulsion was maintained under continuous magnetic stirring (300 rpm) at room temperature until complete solvent evaporation resulted in a stable microparticle suspension.

MPs were characterized by scanning electron microscopy (SEM). The samples were deposited on mica surfaces, mounted on conductive carbon tape, coated with gold, and analyzed using a Tescan Vega3 LMU microscope operating at 15 kV. Protein immobilization analysis was performed in a quartz crystal microbalance with dissipation monitoring (QCM-D; Biolin Scientific AB, Sweden) as previously described.^23^ For CLSM analysis, 10 μL of MPs containing the immobilized protein was mixed with glycerol and placed between glass slides. All analyses were performed on a laser-scanning confocal multiphoton microscope (Nikon A1MP+, Japan) using a 60x objective (NA 1.40).

### 2.5 Nanoparticle Formulation and Characterization

The PHB nanoparticles were prepared via the emulsion-evaporation method as previously reported in ref 27. PHB was first solubilized in chloroform at a concentration of 12.5 mg mL^–1^ and subsequently combined with an aqueous PVA solution (1%, w/v). The resulting mixture was pre-emulsified by vortexing and then subjected to probe sonication (Branson Digital Sonifier, USA) for 1.5 min at 30% amplitude. The organic solvent was subsequently removed under continuous magnetic stirring in a fume hood. Following complete chloroform evaporation, the nanoparticle suspension was brought to a final volume of 10 mL with ultrapure water.

The samples were diluted in Tris-HCl buffer and characterized by dynamic light scattering (DLS) and electrophoretic light scattering using a Zetasizer Nano ZS instrument (Malvern). The NP morphology was examined by transmission electron microscopy (TEM) and cryogenic transmission electron microscopy (cryo-TEM).^23^

### 2.6 Protein Immobilization

All recombinant proteins were previously diluted to 50 μg.mL^–1^ using Tris-HCl buffer (50 mM, pH 8.0) containing 150 mM NaCl. For microparticle immobilization experiments, fixed protein volumes were incubated with increasing PHB microparticle concentrations to generate distinct protein-to-polymer ratios: a fixed 10 μL volume of all proteins (50 μg.mL^–1^) was added to MP volumes of 10, 100, and 1000 μL. Each mixture had a total PHB concentration of 2.5 mg.mL^–1^, corresponding to ratios of protein (mg.mL^–1^)/PHB (mg.mL^–1^) = 20, 2, and 0.2, respectively. The samples were maintained under gentle stirring for 30 min at room temperature. Next, the samples were centrifuged at 15,093 × *g* for 30 s, the supernatant was carefully removed, and the unbound proteins were quantified via fluorescence using a BioTek Synergy, Agilent, with excitation at 428 nm and emission at 528 nm. For protein immobilization on the NP, 50 μL of NP was incubated with 50 μL of each construct (50 μg.mL^–1^) for 1 h at 30 °C. Next, the samples were centrifuged at 15,093 × *g* for 5 min, and the supernatant was quantified for unbound protein via fluorimetry as previously described.^20^

### 2.7 Circular Dichroism Analysis

Circular dichroism analysis was performed using a nitrogen-flushed OLIS DSM20 UV/vis spectropolarimeter (OLIS, USA). All analyses were carried out at the Cedro Beamline, at the Brazilian Synchrotron Laboratory. Prior to analysis, all proteins were diluted to 50 μg mL^–1^ in Tris-HCl buffer, pH 8.0, dialyzed against ultrapure water (12 kDa MWCO), and analyzed in the far-UV region (190–250 nm). The NP or NP-containing immobilized proteins were also dialyzed (100 kDa MWCO) against ultrapure water prior to analysis. Measurements were taken at 37 °C with an integration time = 1 s, a 0.2 mm path-length quartz cell, and bandwidth = 1 nm. Baseline correction was performed by subtracting the buffer background, and the resulting data were processed with CDToolX and BeStSel.^28^

### 2.8 Scanning Near-Field Optical Microscopy

Near-field infrared characterization was carried out by scattering-type scanning near-field optical microscopy (s-SNOM) at the Imbuia beamline of the Brazilian Synchrotron Light Laboratory (LNLS). Samples were prepared by placing a 3 μL aliquot onto a gold substrate, followed by overnight drying. Measurements were conducted under an N_2_ atmosphere with a neaSNOM platform (Neaspec GmbH). Surface topography was simultaneously recorded in tapping mode using PtIr-coated AFM probes (Arrow PtIr, NanoWorld AG) operated at approximately 250 kHz. For spectroscopic acquisition, the system was coupled to a mercury cadmium telluride (MCT) detector with a 50 × 50 μm^2^ active area (InfraRed Associates Inc.). Spectral data were collected at 6 cm^–1^ resolution, with each interferogram comprising 2,048 sampling points and a 20 ms integration time at each point. At each analyzed position, 30 independently acquired interferograms were Fourier transformed and averaged to generate the corresponding near-field spectrum.

### 2.9 Cellular Internalization Assay

The interaction of the engineered NP:protein constructs with mammalian cells was investigated using MCF7 human breast cancer cells. MCF7 cultures were maintained in RPMI 1640 containing 10% fetal bovine serum (FBS) and 10 μg mL^–1^ insulin. Cells were kept at 37 °C in a humidified incubator equilibrated with 5% CO_2_.

For the time-dependent uptake experiments, 5 × 10^4^ cells were seeded per well onto round coverslips in 24-well plates and allowed to adhere for 18 h. The culture medium was then replaced with medium containing the corresponding NP preparation at 500 μg mL^–1^. Prior to cell treatment, the NP suspensions were dialyzed using a 100 kDa MWCO membrane (SpectraPor), and the nanoparticles were fluorescently labeled with Nile Red. Cell exposure was carried out for 1, 3, 6, 24, or 48 h. At the respective end points, the cultures were rinsed with 1× PBS and fixed in 2% (w/v) paraformaldehyde. Following fixation, the cells were washed with PBS, permeabilized with 0.01% (w/v) saponin, and washed once more. Nuclear counterstaining and mounting were performed using DAPI-containing Fluoromount-G.

A separate set of MCF7 cultures was prepared on glass-bottom dishes (CellView, Greiner) for confocal laser scanning microscopy (CLSM). Cells were exposed to NP or the respective purified proteins at 100 μg mL^–1^. During the experiment, cultures were maintained at 37 °C and 5% CO_2_. Imaging was conducted on a Nikon A1MP+ multiphoton confocal system (Nikon, Japan) fitted with a 60× oil-immersion objective (NA 1.40). Image acquisition was controlled through NIS-Elements 4.20 (Nikon), whereas subsequent processing and quantitative image analysis were carried out in ImageJ. Cell-associated fluorescence was independently quantified by flow cytometry. For these experiments, MCF7 cells were plated in 24-well plates at 1 × 10^6^ cells per well. After an 18 h attachment period, cultures received 500 μg mL^–1^ of the corresponding NP preparation and were incubated for 1, 3, 6, 24, or 48 h. Measurements were performed on a FACSCalibur flow cytometer (BD Biosciences, USA). A total of 10,000 events was recorded for each condition. The resulting fluorescence measurements were normalized against the appropriate controls and reported as relative percentages.

### 2.10 Statistical Analysis

All statistical calculations were performed in GraphPad Prism (version 6.0). The statistical approach was selected according to the number of experimental groups being compared. Pairwise comparisons were assessed with Student’s *t*-test, while data sets comprising three or more groups were evaluated by one-way ANOVA with subsequent post hoc testing, when required. Differences were considered statistically significant when *p* < 0.05. The levels of significance displayed in the figures were defined as follows: *p* < 0.05 (*), p < 0.01 (**), and *p <* 0.001 (***).

## 3 Results and Discussion

### 3.1 PHB and sfGFP–Linker–SBD Constructs

The PHB used to prepare MP was initially characterized by NMR in CDCl_3_, which displayed characteristic ^13^C chemical shifts centered at 21.1, 42.2, 69.0, and 170 ppm, corresponding to −CH_3_, −CH_2_, −CH, and −C=O resonances, respectively. The ^1^H chemical shifts were observed at 1.3, 2.57, and 5.29 ppm, corresponding to −CH_3_, −CH_2_, and −CH resonances, respectively (Figure 1A and B), as previously reported.^29^ The material is polydisperse, with Đ ∼1.6, *M*_w_ ∼640,000 g·mol^–1^, and *M*_n_ ∼390,000 g·mol^–1^ (Figure S1).

**Figure 1:**
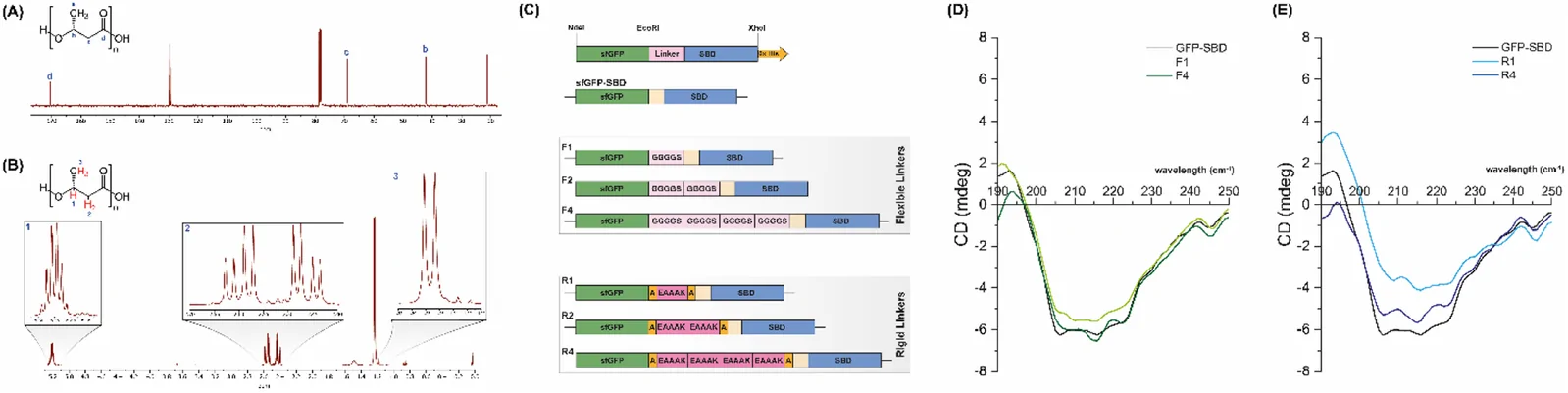
PHB and fusion protein construct characterization. ^13^C (A) and ^1^H (B) NMR spectra of PHB performed in CDCl_3_, containing peak assignments at the insets. Schematic representation of sfGFP–linker–SBD constructions (C); circular dichroism profile of sfGFP-SBD, F1, and F4 (D) and sfGFP-SBD, R1, and R4 (E).

The schematic representations and structural models of each fusion construct are shown in Figure 1C, highlighting the disposition of the sfGFP, linker, and SBD units. SDS-PAGE (Figure S1) characterization revealed distinct electrophoretic profiles: sfGFP presents *M*_w_ ∼30 kDa, whereas those containing the SBD as well as the linker groups exhibited reduced electrophoretic mobility, with *M_w_* ∼40 kDa, indicating the presence of the SBD domain—*M*_w_ ∼10 kDa.^30^ Although most samples also displayed untagged sfGFP fractions, the full proteins were obtained in higher amounts. SAXS data revealed an average radius in solution of 3.6 nm—similar to sfGFP.^31^ Moreover, the sfGFP fluorescence emission and excitation spectra were not modified by either the SBD or the linker groups (Figure S2).

Structural investigation (Figure 1D and E) revealed that all constructs possess an increased amount of unordered structures compared to sfGFP.^32^ In particular, sfGFP-SBD exhibits ∼48% of structural disordered elements due to the contribution of the SBD domain as well as the original PHA-depolymerase linker (GSTTKA). With increasing linker flexibility from F1 to F4, the α-helix, β-sheet, and turn contents decrease, whereas the participation of disordered structures increases from 46 to 66%. Possibly, as the segmental freedom increases with (GGGGS) repeating units, the CD data indicate an average spectrum that reflects distinct or shifting conformations in solution.^33^ Conversely, from R1 to R4, the α-helical contribution increases from approximately 5 to 23% with increasing numbers of (EAAAK)_n_ linker units, whereas the proportion of disordered structures remains lower than that observed for the corresponding flexible-linker constructs. This trend is consistent with the intrinsic α-helical propensity of EAAAK-based linkers and indicates that linker composition contributes directly to the distinct secondary-structure profiles of the R-series.

### 3.2 Linker Architecture Tunes Adsorption and Immobilization on PHB Microparticles

The PHB MPs exhibit predominantly spherical morphology with pores and irregular polymer distribution within hollow compartments (Figure 2A). To determine the adsorption behavior of the fusion constructs, the MPs were deposited over QCM-D sensors (Figures S3, S4) and each protein sample was independently analyzed, as schematically represented in Figure 2B. The frequency shift after the rinsing step revealed that the wild-type sfGFP displays poor deposition compared to sfGFP-SBD, in accordance with previous observations.^20^ However, as the flexibility of the linker group increases, protein immobilization over the PHB MPs decreases to the same range as sfGFP (Figure 2C). Conversely, increased linker rigidity leads to higher protein adsorption, with R4 displaying the same amount as sfGFP-SBD. Although the QCM-D results suggested that sfGFP-SBD and R4 exhibited higher immobilization efficiency than the other samples, these experimental conditions reflect sample behavior under a continuous 100 μL.min^–1^ flow. The water contact angle of each surface after the deposition process showed that only the sfGFP-SBD, R2, and R4 samples significantly reduced the contact angle—from 49 ± 2° for PHB MPs vs ∼20° for all the latter samples (Figure S3C)—thereby corroborating higher protein-immobilized amounts. These results suggest that the sfGFP domain may interfere with the SBD unit, and vice versa, thereby preventing binding to PHB; the increased segmental flexibility may enable close-proximity states between the two units, thereby hindering the SBD from engaging in preferential hydrophobic interactions with the polymeric interface.^34^

**Figure 2:**
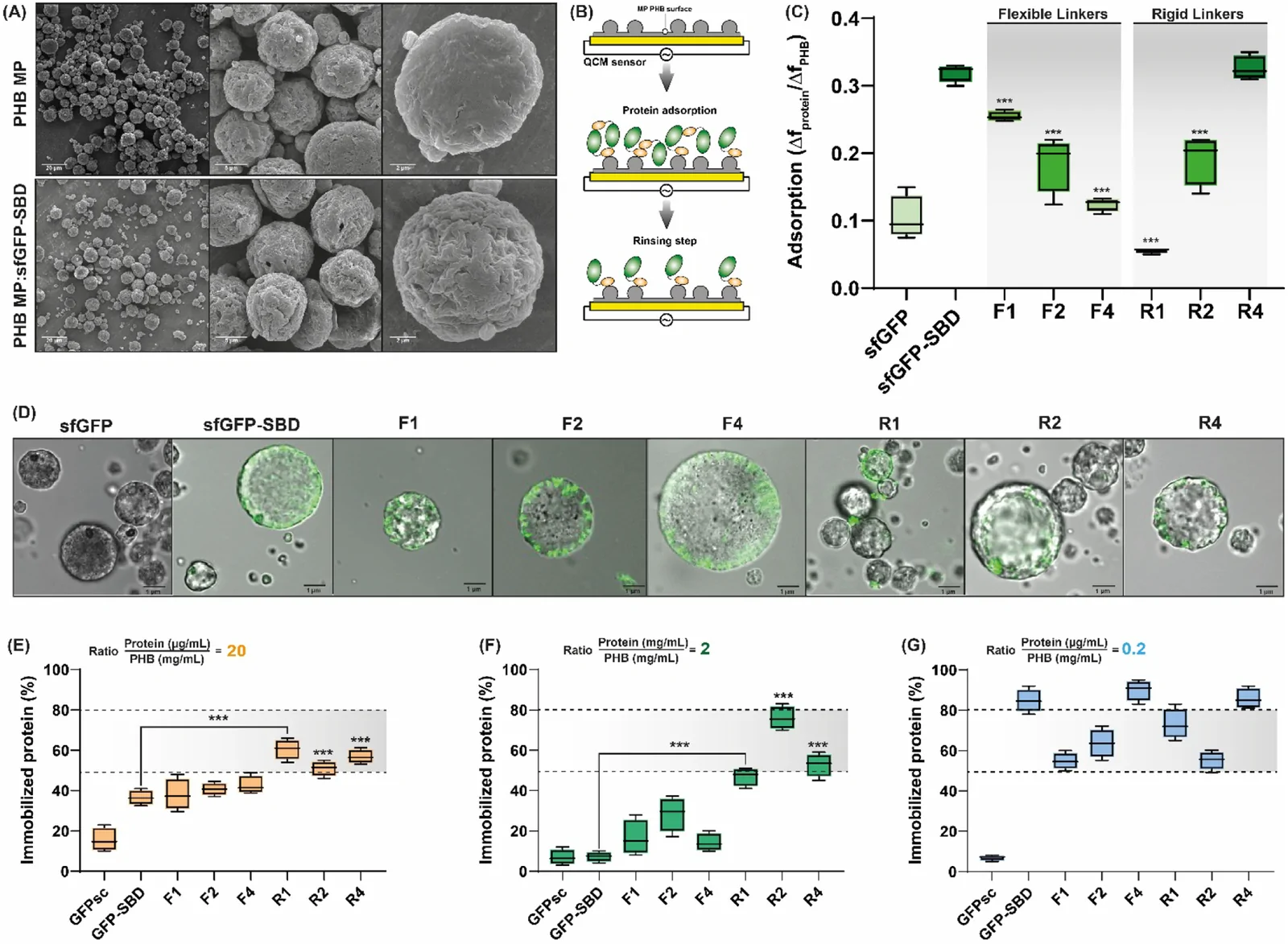
Microparticle characterization and binding assay with all fusion proteins. SEM images of bare and sfGFP-SBD-coated microparticles (A). Scale bars of 20, 5, and 2 μm. Schematic representation of QCM-D experiments (B) with respective protein adsorption (C). CLSM images of differential interference contrast and fluorescence merged channels for MPs after protein immobilization (D). Scale bar of 1 μm. Protein quantification assay for different protein:MP ratios (E–G). The data are presented as mean ± SD; statistical analyses are indexed as ***p < 0.001.

The protein adsorption over PHB MPs was corroborated by CLSM images (Figure 2C and Figure S5). All fusion proteins generated green fluorescent MPs with localized, concentration-dependent intensity at the polymer–water interface, following the rough edges. The proteins were not removed by washing cycles, and only the untagged sfGFP produced MPs with reduced fluorescence intensity. To quantify the amount of immobilized protein, the supernatant of each sample was analyzed by fluorimetry: as shown in Figure 2E–G, the available surface area, i.e., the protein:PHB ratio, influences the extent of protein binding. At the lowest PHB surface area, ratio = 20, the constructs presenting rigid linkers displayed higher immobilization amounts compared to the other samples. As the available PHB surface increases (ratio = 2), R1, R2, and R4 also show increased immobilized content, whereas the sfGFP-SBD and all of the flexible linker-containing constructs reveal inferior values. At the highest PHB surface area (ratio = 0.2), all samples exhibited >50% immobilization efficiency.

Overall, these results demonstrate that the linker architecture influences the immobilization efficiency of the fusion constructs on PHB interfaces. The higher binding efficiencies observed for the rigid-linker constructs, particularly R2 and R4, are consistent with a model in which reduced interdomain conformational freedom may favor configurations that are more compatible with the SBD’s interaction with the PHB surface. Conversely, the greater conformational freedom provided by the flexible (GGGGS)_n_ linkers may permit a broader ensemble of interdomain arrangements, some of which could be less favorable for the surface interaction.

These findings extend our previous observations regarding SBD-mediated immobilization on PHB interfaces. In our earlier studies, the sfGFP-SBD construct established the feasibility of using the substrate-binding domain to promote protein association with PHB micro- and nanostructures.^20,23^ However, those studies did not investigate how the molecular architecture connecting the reporter and binding domains influences interfacial performance. The systematic linker comparison performed here reveals that increasing interdomain separation alone does not necessarily improve PHB binding. Instead, linker rigidity emerges as an important design parameter: constructs containing the rigid (EAAAK)_n_ linkers, particularly R2 and R4, consistently displayed higher immobilization efficiencies than their flexible counterparts under the evaluated conditions. Thus, the present results advance beyond demonstrating SBD-mediated PHB binding by showing that the architecture of the interdomain linker can modulate the efficiency of this interaction. These observations establish a structure–interfacial–function relationship that may guide the rational design of fusion proteins for the functionalization of polyester-based micro- and nanostructures.

### 3.3 Protein Immobilization on PHB Nanoparticles Perturbs the Construct Structure in a Linker-Dependent Manner

The PHB NP exhibited spherical morphology, with a hydrodynamic diameter of 190 ± 75 nm, PDI < 0.25, a ζ-potential of −10 ± 0.1 mV, and a bulky structure as evidenced by cryo-TEM (Figure 3A-C, Table S2). After mixing the NP with the proteins and submitting the samples to 100 kDa MWCO dialysis, there was no significant shift in size distribution or morphology. The binding assay was performed using a protein:NP ratio = 0.4 and showed that the untagged sfGFP protein has the lowest immobilization efficiency, whereas sfGFP-SBD, R2, and R4 stand out with immobilization efficiencies between 55 and 63% (Figure 3D). Although the PHB mass is lower compared to the experimental conditions evaluated for the MP, the surface area increases as the system is formulated at the nanoscale, which highlights the better performance of R2 and R4 in most of the evaluated conditions. Nonetheless, a significant fraction (∼40%) remains unbound, which hinders the SBD from binding to the PHB interface. The ζ-potential analysis corroborates the previous results by showing that sfGFP-SBD, R2, and R4 cause a significant decrease in the NP’s electrical charge at the slipping plane, thereby suggesting a more efficient association compared to the other samples (Figure S6, Table S2).

**Figure 3:**
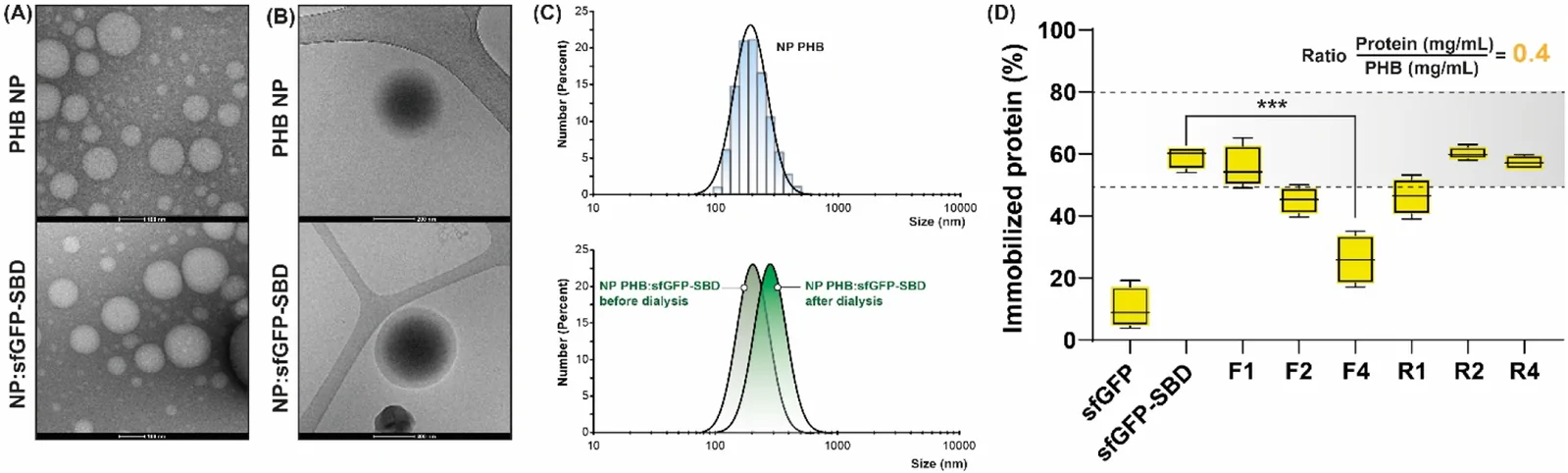
Nanoparticle characterization and binding assay with all fusion proteins. TEM (A) and cryo-TEM (B) of PHB NPs with and without sfGFP-SBD. Scale bars of 100 and 200 nm, respectively. Comparison of NP size before and after protein incubation and dialysis (C). Protein quantification assay (D). The data is plotted as mean ± SD; statistical analyses are indexed as ***p < 0.001.

Protein adsorption was further confirmed by localized s-SNOM analysis. Accordingly, the NPs presented a characteristic sharp band at 1,737 cm^–1^ corresponding to the PHB carbonyl (C=O) stretching, along with several signals from 1,000 to 1,300 cm^–1^ that indicate the presence of ester group C–O bond stretching.^29^ The NPs containing immobilized proteins also showed the carbonyl band at 1,745 cm^–1^ as well as other bands originally displayed by the PHB NPs that are absent in the respective pure protein spectra (Figure S6). Moreover, after the protein adsorption process and dialysis, the NP IR spectra displayed overlapping PHB and protein signals with distinct intensities throughout the surface, which confirm protein immobilization over the NPs (Figure 4A–F). In particular, the NP:sfGFP-SBD and NP:R4 samples showed bands at 1,635, 1,660, and 1,550 cm^–1^ that indicate the presence of amide I and II, typical of proteins, and absence from the PHB spectra.^35^

**Figure 4:**
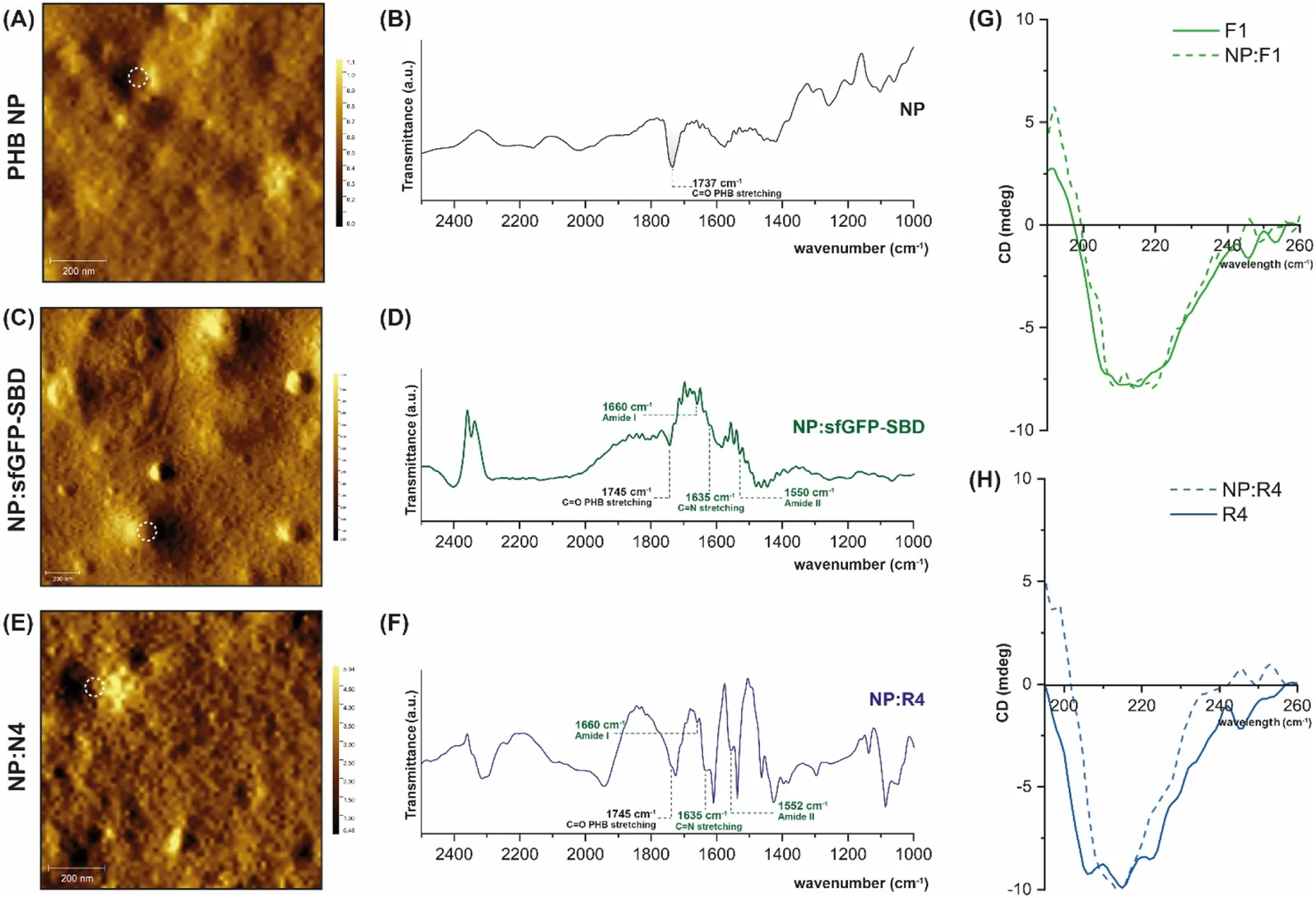
Nanoparticle and protein interaction analysis. s-SNOM-derived AFM amplitude images and respective IR spectra of NPs (A and B), NP:sfGFP-SBD (C and D), and NP:R4 (E and F). The IR region is highlighted by a white circle. Circular dichroism spectra of F1 and NP:F1 (G) and R4 and NP:R4 (H).

To analyze the overall impact of immobilization on the protein’s secondary structure, we performed CD on the NP–protein solution after dialysis. As shown in Figure 4G, the construct containing the short flexible linker F1 reveals a discrete signal shift after attaching to the NP, suggesting limited rearrangement upon interacting. It is interesting to observe that, after the immobilization procedure, the SBD-containing protein fraction remained predominantly associated with the NP, as shown for the NP:R1 sample (Figure S6B): native polyacrylamide gel electrophoresis reveals that only the untagged protein fraction penetrates the gel, whereas the SBD-tagged constructs remain within the NP fraction, which is too large to migrate through the gel mesh. In addition, the NP:F1 CD spectra measured from 20 to 90 °C indicate that the F1 protein might undergo conformational changes upon immobilization on the NP, as evidenced by a higher melting temperature (*T*_m_) of 61 ± 2.4° vs 52 ± 2.4° for the free protein (Figure S7).

The NP:R4 sample, however, shows significant differences compared to R4, including an increased signal at ∼195 nm, a decreased amplitude at ∼210–220 nm, and altered patterns between 230 and 250 nm (Figure 4H). These changes indicate that association with the PHB NP interface modifies the overall conformational ensemble of the fusion construct.

### 3.4 Dual-Fluorescence Tracking of NP Internalization and Real-Time Comparison of Free vs NP-Immobilized Protein Uptake in MCF7 Cells

To determine the feasibility of tracking the sfGFP fluorescence signal during cellular internalization assays, we previously labeled the PHB NPs with Nile Red, incubated them with R4, and dialyzed them to remove loosely bound proteins. During CLSM and flow cytometry, we monitored the fluorescence intensity in both channels after 1, 3, 6, 24, and 48 h of incubation.

The results shown in Figure 5 reveal that, after 1 h, the NPs are irregularly distributed throughout the cell cytoplasm by observing both red and green intracellular fluorescent spots, while the latter presents significantly lower intensity compared to Nile Red with equal localization; these observations were corroborated by flow cytometry that confirmed NP uptake via both channels with similar median values. At t = 3 h, it is possible to distinguish several endosomes labeled by NP accumulation, as similarly observed for poly­(lactic acid)-*b*-poly­(ethylene glycol)^36^ and PHB nanostructures,^23^ though PHB MPs display significantly lower internalization efficiency as a result of their larger size range.^20^ Nonetheless, after 6 h, the fluorescent distribution became more diffuse and individual vesicular structures were less clearly distinguishable.^37^ At t = 24 h, the PHB signal decreases significantly, indicating that the NP internalization rate declines and the polymeric system undergoes degradation. However, in 6 and 24 h, it is possible to notice localized sfGFP intensities without Nile Red overlapping, possibly as a result of the protein’s stability in the acidic environment^38^ while the polymer is hydrolyzed. After 48 h, the red and green fluorescence is colocalized along the cytosol, though several sfGFP signals may be individually visualized in the CLSM merged image, which suggests the presence of remaining protein as well as recently internalized NPs. The amount of NP uptake follows a similar profile to sfGFP, thereby demonstrating the potential of R4 to enable tracking and determination of NP accumulation.

**Figure 5:**
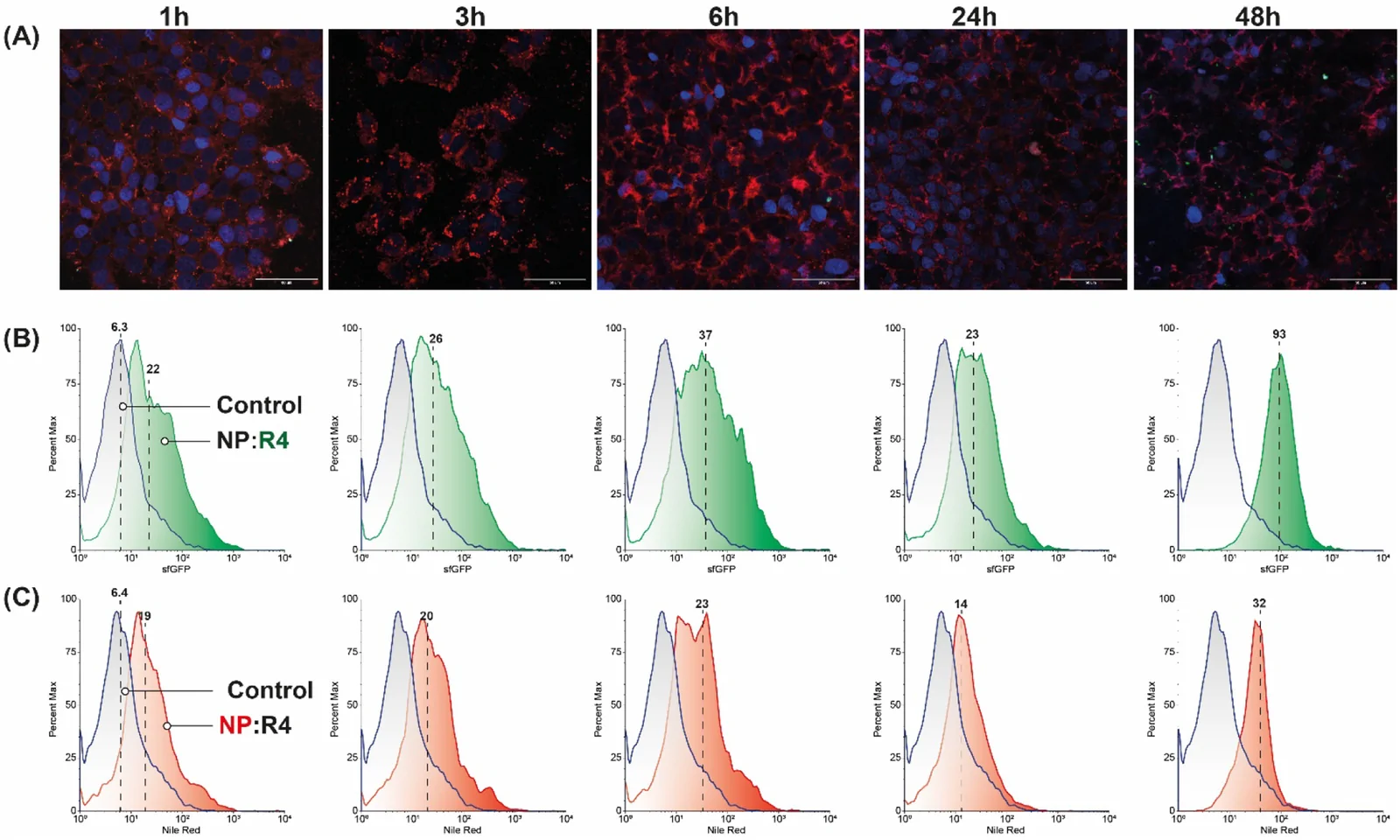
Nanoparticle internalization in MCF7. Confocal laser scanning microscopy images as visualized at the merged channel (A) and flow cytometry profiles of MCF7 cells treated with a 500 μg.mL^–1^ solution of NP:R4 (B) and NP:R4 labeled with Nile Red (C) from 1 to 48 h.

To evaluate the influence of our nanoplatforms on protein distribution *in vitro*, we monitored the sfGFP fluorescence channel at different time intervals after adding free or NP-containing immobilized proteins to the culture medium. As shown in Figure 6, free proteins accumulate around the cells and form extracellular aggregates without increasing cytosolic fluorescence intensity from t = 0 to t = 10 h. Afterward, the number of particle systems increases on the cell surface and in the extracellular space, whereas a discrete number undergoes internalization. Alternatively, NP:sfGFP-SBD and NP:R4 prevented protein aggregation at t = 0 and increased the amount of protein delivered into cells from t = 0.5 to 24 h, suggesting a time-dependent uptake mechanism. Both nanosystems avoided protein accumulation at the cellular edges and enabled monitoring of ∼200 nm NP accumulation and the dynamic motion of cytosolic endosomes (Figure 6B and D) over 24 h.

**Figure 6:**
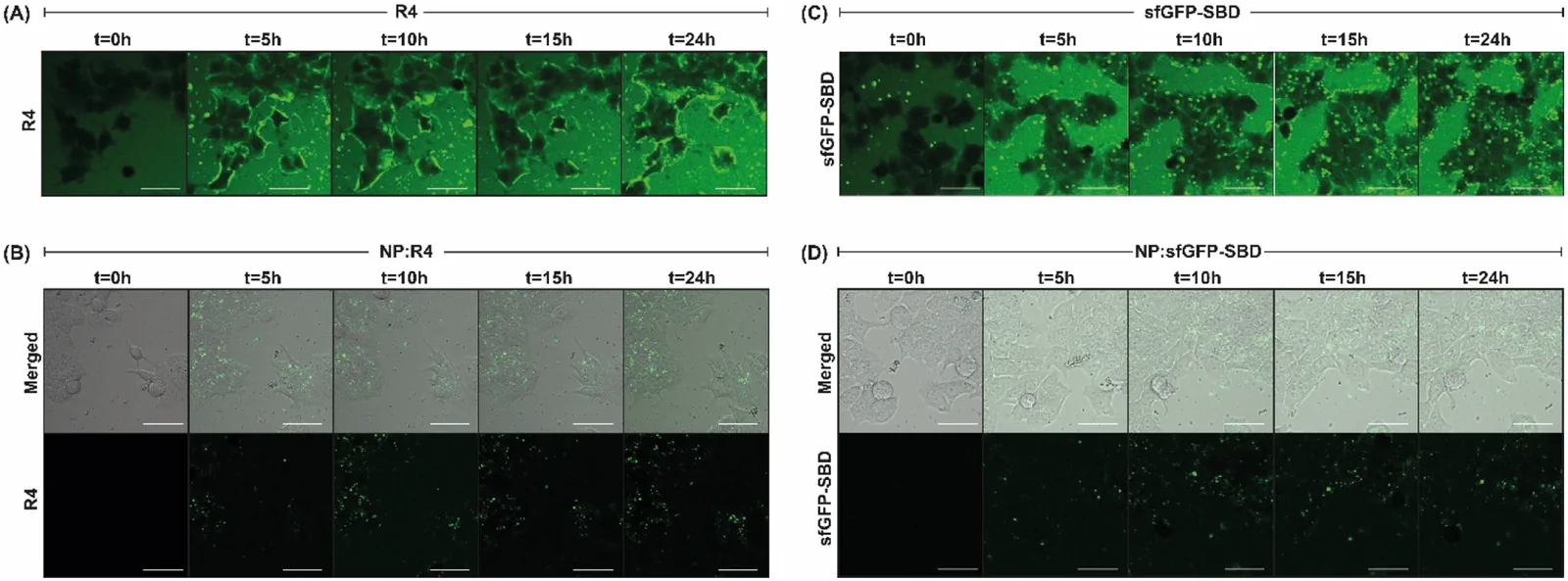
Timelapse of nanoparticle and free protein internalization in MCF7. Fluorescence channel for pure R4 and sfGFP-SBD added to MCF7 cells (A and C). Differential interference contrast and fluorescence channels for NP:R4 and NP:sfGFP-SBD (B and D) from 0 to 24 h. Scale bar: 50 μm.

The persistence of the sfGFP-associated signal throughout the cellular uptake experiment is also relevant, considering the structural changes detected by CD after R4 immobilization. Although these measurements do not provide quantitative information on fluorescence quantum yield or photobleaching kinetics, they demonstrate that nanoparticle association does not abolish sfGFP reporter functionality and that detectable fluorescence is retained under the cellular conditions investigated for up to 48 h.

## 4 Conclusions

In this study, we investigated the effects of flexible and rigid linkers with varying extensions on the binding of the sfGPF-SBD fusion protein to the PHB interfaces. The constructs linked by rigid sequences with n = 2 and n = 4 exhibited a higher PHB adsorption capacity across all analyzed conditions (more than 50%) than the MP and NP. Along with structural data, our results suggest that the reduced segmental mobility conferred by the rigid linkers may contribute to configurations that are more favorable for the SBD-mediated interaction with PHB interfaces. The stable association provided by the constructs and PHB NPs enabled dual-fluorescence tracking of nanosystem internalization by MCF7 cells. In addition, the nanosystems decorated with R4 increased localized cellular delivery, which will be explored along with the development of a new generation of nanovaccines.

## Supplementary Material



## Notes

DNA sequences and protein characterization, morphological characterization of microparticles and protein binding over a coated QCM-D sensor, and nanoparticle characterization and protein binding (PDF)
