# Supplementary material for: Tailoring Fusion Proteins for Interfacial Nanoparticle Functionalization: Toward Fluorescence-Guided Imaging
Source: ACS Omega. 2026 Oct 13;11(42):65865–74. doi: 10.1021/acsomega.6c09485 (PMC13718197; doi:10.1021/acsomega.6c09485)
Supplement: Supplementary file 1 [file ao6c09485_si_001.pdf]

Supporting Information

Tailoring fusion proteins for interfacial nanoparticle functionalization: towards fluorescence-guided imaging

Ryu Masaki[1,†], Julia T. Lucena[1,†], Maritza A. Todo Bom[1], Isabela P. Dias[1], Mariana Vodiani[1], Giovanna Furman[1], Joslaine Jacumazo[2], Rilton A. de Freitas[2], Guinther Kellerman[3], Maísa Colodel[3], Luiz M. de L. Faria[4], Gabriela C. Cardoso[5], Edneia A. S. Ramos[5], Lindomar J. C. Albuquerque[6], Genilza da Silva Mello[7], Camila de Almeida Salvego[1], Breno C. B. Beirão[5], Edileusa M. Gerhardt[1], Emanuel Maltempi de Souza[1], Marcelo Müller-Santos[1,*], Guilherme F. Picheth[1,*]

[1]Department of Biochemistry and Molecular Biology, Post-graduate Program in Biochemistry, Federal University of Paraná, Curitiba, PR, 81531-980, Brazil.

[2]Department of Pharmacy, Federal University of Paraná, Curitiba, PR, 80210-170, Brazil.

[3]Department of Physics, Federal University of Paraná, Curitiba, PR, 81531-980, Brazil.

[4]Department of Chemistry and Biology, Federal Technological University of Paraná, Curitiba, PR, 81280-340, Brazil.

[5]Post-graduate Program in Microbiology, Parasitology and Pathology, Federal University of Paraná, Curitiba, PR, 81531-980, Brazil

[6]Brazilian Synchrotron Light Laboratory, Brazilian Center for Research in Energy and Materials, Campinas, SP, 13083-100, Brazil.

[7]Araguaia University Center, CEP 78.603-209, Barra do Garças, MT, 78603-209, Brazil.

Table S1. DNA sequences for all fusion constructs

Sample	DNA sequence
sfGFP-SBD	CATATGAAAGAAACCGCTGCTGCTAAATTCGAACGCCAGCACATGGACAGCCCAGATCTGGGTACCCTGGTGCCACGCGGTTCCATGCGT<br>AAAGGCGAAGAGCTGTTCACTGGTGTCGTCCCTATTCTGGTGGAACTGGATGGTGATGTCAACGGTCATAAGTTTTCCGTGCGTGGCGAG<br>GGTGAAGGTGACGCAACTAATGGTAAACTGACGCTGAAGTTCATCTGTACTACTGGTAAACTGCCGGTACCTTGGCCGACTCTGGTAACG<br>ACGCTGACTTATGGTGTTCAGTGCTTTGCTCGTTATCCGGACCATATGAAGCAGCATGACTTCTTCAAGTCCGCCATGCCGGAAGGCTATG<br>TGCAGGAACGCACGATTTCCTTTAAGGATGACGGCACGTACAAAACGCGTGCGGAAGTGAAATTTGAAGGCGATACCCTGGTAAACCGCA<br>TTGAGCTGAAAGGCATTGACTTTAAAGAAGACGGCAATATCCTGGGCCATAAGCTGGAATACAATTTTAACAGCCACAATGTTTACATCACC<br>GCCGATAAACAAAAAAATGGCATTAAAGCGAATTTTAAAATTCGCCACAACGTGGAGGATGGCAGCGTGCAGCTGGCTGATCACTACCAG<br>CAAAACACTCCAATCGGTGATGGTCCTGTTCTGCTGCCAGACAATCACTATCTGAGCACGCAAAGCGTTCTGTCTAAAGATCCGAACGAGA<br>AACGCGATCATATGGTTCTGCTGGAGTTCGTAACCGCAGCGGGCATCACGCATGGTATGGATGAACTGTACAAAGGATCTACCACCAAAT<br>CTGCTTTCACCTGCACCGCTACCACCGCTTCTAACTACGCTCACGTTCAGGCTGGTCGTGCTCACGACTCTGGTGGTATCGCTTACGCTAA<br>CGGTTCTAACCAGTCTATGGGTCTGGACAACCTGTTCTACACCTCTACCCTGGCTCAGACCGCTGCTGGTTACTACATCGTTGGTAACTGC<br>CCGCTCGAG
sfGFP-F1-SBD	CATATGCGTAAAGGCGAAGAGCTGTTCACTGGTGTCGTCCCTATTCTGGTGGAACTGGATGGTGATGTCAACGGTCATAAGTTTTCCGTGC<br>GTGGCGAGGGTGAAGGTGACGCAACTAATGGTAAACTGACGCTGAAGTTCATCTGTACTACTGGTAAACTGCCGGTACCTTGGCCGACTC<br>TGGTAACGACGCTGACTTATGGTGTTCAGTGCTTTGCTCGTTATCCGGACCACATGAAGCAGCATGACTTCTTCAAGTCCGCCATGCCGGA<br>AGGCTATGTGCAGGAACGCACGATTTCCTTTAAGGATGACGGCACGTACAAAACGCGTGCGGAAGTGAAATTTGAAGGCGATACCCTGGT<br>AAACCGCATTGAGCTGAAAGGCATTGACTTTAAAGAAGACGGCAATATCCTGGGCCATAAGCTGGAATACAATTTTAACAGCCACAATGTT<br>TACATCACCGCCGATAAACAAAAAAATGGCATTAAAGCGAATTTTAAAATTCGCCACAACGTGGAGGATGGCAGCGTGCAGCTGGCTGATC<br>ACTACCAGCAAAACACTCCAATCGGTGATGGTCCTGTTCTGCTGCCAGACAATCACTATCTGAGCACGCAAAGCGTTCTGTCTAAAGATCC<br>GAACGAGAAACGCGATCACATGGTTCTGCTGGAGTTCGTAACCGCAGCGGGCATCACGCATGGTATGGATGAACTGTACAAAGAATTCGG<br>TGGTGGTGGTTCTACCACCAAATCTGCTTTCACCTGCACCGCTACCACCGCTTCTAACTACGCTCACGTTCAGGCTGGTCGTGCTCACGA<br>CTCTGGTGGTATCGCTTACGCTAACGGTTCTAACCAGTCTATGGGTCTGGACAACCTGTTCTACACCTCTACCCTGGCTCAGACCGCTGCT<br>GGTTACTACATCGTTGGTAACTGCCCGCTCGAG
sfGFP-F2-SBD	CATATGCGTAAAGGCGAAGAGCTGTTCACTGGTGTCGTCCCTATTCTGGTGGAACTGGATGGTGATGTCAACGGTCATAAGTTTTCCGTGC<br>GTGGCGAGGGTGAAGGTGACGCAACTAATGGTAAACTGACGCTGAAGTTCATCTGTACTACTGGTAAACTGCCGGTACCTTGGCCGACTC<br>TGGTAACGACGCTGACTTATGGTGTTCAGTGCTTTGCTCGTTATCCGGACCACATGAAGCAGCATGACTTCTTCAAGTCCGCCATGCCGGA<br>AGGCTATGTGCAGGAACGCACGATTTCCTTTAAGGATGACGGCACGTACAAAACGCGTGCGGAAGTGAAATTTGAAGGCGATACCCTGGT<br>AAACCGCATTGAGCTGAAAGGCATTGACTTTAAAGAAGACGGCAATATCCTGGGCCATAAGCTGGAATACAATTTTAACAGCCACAATGTT<br>TACATCACCGCCGATAAACAAAAAAATGGCATTAAAGCGAATTTTAAAATTCGCCACAACGTGGAGGATGGCAGCGTGCAGCTGGCTGATC<br>ACTACCAGCAAAACACTCCAATCGGTGATGGTCCTGTTCTGCTGCCAGACAATCACTATCTGAGCACGCAAAGCGTTCTGTCTAAAGATCC<br>GAACGAGAAACGCGATCACATGGTTCTGCTGGAGTTCGTAACCGCAGCGGGCATCACGCATGGTATGGATGAACTGTACAAAGAATTCGG<br>TGGTGGTGGTTCTGGTGGTGGTGGTTCTACCACCAAATCTGCTTTCACCTGCACCGCTACCACCGCTTCTAACTACGCTCACGTTCAGGCT<br>GGTCGTGCTCACGACTCTGGTGGTATCGCTTACGCTAACGGTTCTAACCAGTCTATGGGTCTGGACAACCTGTTCTACACCTCTACCCTGG<br>CTCAGACCGCTGCTGGTTACTACATCGTTGGTAACTGCCCGCTCGAG
sfGFP-F3-SBD	CATATGCGTAAAGGCGAAGAGCTGTTCACTGGTGTCGTCCCTATTCTGGTGGAACTGGATGGTGATGTCAACGGTCATAAGTTTTCCGTGC<br>GTGGCGAGGGTGAAGGTGACGCAACTAATGGTAAACTGACGCTGAAGTTCATCTGTACTACTGGTAAACTGCCGGTACCTTGGCCGACTC<br>TGGTAACGACGCTGACTTATGGTGTTCAGTGCTTTGCTCGTTATCCGGACCACATGAAGCAGCATGACTTCTTCAAGTCCGCCATGCCGGA<br>AGGCTATGTGCAGGAACGCACGATTTCCTTTAAGGATGACGGCACGTACAAAACGCGTGCGGAAGTGAAATTTGAAGGCGATACCCTGGT<br>AAACCGCATTGAGCTGAAAGGCATTGACTTTAAAGAAGACGGCAATATCCTGGGCCATAAGCTGGAATACAATTTTAACAGCCACAATGTT<br>TACATCACCGCCGATAAACAAAAAAATGGCATTAAAGCGAATTTTAAAATTCGCCACAACGTGGAGGATGGCAGCGTGCAGCTGGCTGATC<br>ACTACCAGCAAAACACTCCAATCGGTGATGGTCCTGTTCTGCTGCCAGACAATCACTATCTGAGCACGCAAAGCGTTCTGTCTAAAGATCC<br>GAACGAGAAACGCGATCACATGGTTCTGCTGGAGTTCGTAACCGCAGCGGGCATCACGCATGGTATGGATGAACTGTACAAAGAATTCGG<br>TGGTGGTGGTTCTGGTGGTGGTGGTTCTGGTGGTGGTGGTTCTGGTGGTGGTGGTTCTACCACCAAATCTGCTTTCACCTGCACCGCTAC<br>CACCGCTTCTAACTACGCTCACGTTCAGGCTGGTCGTGCTCACGACTCTGGTGGTATCGCTTACGCTAACGGTTCTAACCAGTCTATGGGT<br>CTGGACAACCTGTTCTACACCTCTACCCTGGCTCAGACCGCTGCTGGTTACTACATCGTTGGTAACTGCCCGCTCGAG
sfGFP-R1-SBD	CATATGCGTAAAGGCGAAGAGCTGTTCACTGGTGTCGTCCCTATTCTGGTGGAACTGGATGGTGATGTCAACGGTCATAAGTTTTCCGTGC<br>GTGGCGAGGGTGAAGGTGACGCAACTAATGGTAAACTGACGCTGAAGTTCATCTGTACTACTGGTAAACTGCCGGTACCTTGGCCGACTC<br>TGGTAACGACGCTGACTTATGGTGTTCAGTGCTTTGCTCGTTATCCGGACCACATGAAGCAGCATGACTTCTTCAAGTCCGCCATGCCGGA<br>AGGCTATGTGCAGGAACGCACGATTTCCTTTAAGGATGACGGCACGTACAAAACGCGTGCGGAAGTGAAATTTGAAGGCGATACCCTGGT<br>AAACCGCATTGAGCTGAAAGGCATTGACTTTAAAGAAGACGGCAATATCCTGGGCCATAAGCTGGAATACAATTTTAACAGCCACAATGTT<br>TACATCACCGCCGATAAACAAAAAAATGGCATTAAAGCGAATTTTAAAATTCGCCACAACGTGGAGGATGGCAGCGTGCAGCTGGCTGATC<br>ACTACCAGCAAAACACTCCAATCGGTGATGGTCCTGTTCTGCTGCCAGACAATCACTATCTGAGCACGCAAAGCGTTCTGTCTAAAGATCC<br>GAACGAGAAACGCGATCACATGGTTCTGCTGGAGTTCGTAACCGCAGCGGGCATCACGCATGGTATGGATGAACTGTACAAAGAATTCGC<br>TGAAGCTGCTGCTAAAGCTACCACCAAATCTGCTTTCACCTGCACCGCTACCACCGCTTCTAACTACGCTCACGTTCAGGCTGGTCGTGCT<br>CACGACTCTGGTGGTATCGCTTACGCTAACGGTTCTAACCAGTCTATGGGTCTGGACAACCTGTTCTACACCTCTACCCTGGCTCAGACCG<br>CTGCTGGTTACTACATCGTTGGTAACTGCCCGCTCGAG
sfGFP-R2-SBD	CATATGCGTAAAGGCGAAGAGCTGTTCACTGGTGTCGTCCCTATTCTGGTGGAACTGGATGGTGATGTCAACGGTCATAAGTTTTCCGTGC<br>GTGGCGAGGGTGAAGGTGACGCAACTAATGGTAAACTGACGCTGAAGTTCATCTGTACTACTGGTAAACTGCCGGTACCTTGGCCGACTC<br>TGGTAACGACGCTGACTTATGGTGTTCAGTGCTTTGCTCGTTATCCGGACCACATGAAGCAGCATGACTTCTTCAAGTCCGCCATGCCGGA<br>AGGCTATGTGCAGGAACGCACGATTTCCTTTAAGGATGACGGCACGTACAAAACGCGTGCGGAAGTGAAATTTGAAGGCGATACCCTGGT<br>AAACCGCATTGAGCTGAAAGGCATTGACTTTAAAGAAGACGGCAATATCCTGGGCCATAAGCTGGAATACAATTTTAACAGCCACAATGTT<br>TACATCACCGCCGATAAACAAAAAAATGGCATTAAAGCGAATTTTAAAATTCGCCACAACGTGGAGGATGGCAGCGTGCAGCTGGCTGATC<br>ACTACCAGCAAAACACTCCAATCGGTGATGGTCCTGTTCTGCTGCCAGACAATCACTATCTGAGCACGCAAAGCGTTCTGTCTAAAGATCC<br>GAACGAGAAACGCGATCACATGGTTCTGCTGGAGTTCGTAACCGCAGCGGGCATCACGCATGGTATGGATGAACTGTACAAAGAATTCGC<br>TGAAGCTGCTGCTAAAGAAGCTGCTGCTAAAGCTACCACCAAATCTGCTTTCACCTGCACCGCTACCACCGCTTCTAACTACGCTCACGTT<br>CAGGCTGGTCGTGCTCACGACTCTGGTGGTATCGCTTACGCTAACGGTTCTAACCAGTCTATGGGTCTGGACAACCTGTTCTACACCTCTA<br>CCCTGGCTCAGACCGCTGCTGGTTACTACATCGTTGGTAACTGCCCGCTCGAG
sfGFP-R4-SBD	CATATGCGTAAAGGCGAAGAGCTGTTCACTGGTGTCGTCCCTATTCTGGTGGAACTGGATGGTGATGTCAACGGTCATAAGTTTTCCGTGC<br>GTGGCGAGGGTGAAGGTGACGCAACTAATGGTAAACTGACGCTGAAGTTCATCTGTACTACTGGTAAACTGCCGGTACCTTGGCCGACTC<br>TGGTAACGACGCTGACTTATGGTGTTCAGTGCTTTGCTCGTTATCCGGACCACATGAAGCAGCATGACTTCTTCAAGTCCGCCATGCCGGA<br>AGGCTATGTGCAGGAACGCACGATTTCCTTTAAGGATGACGGCACGTACAAAACGCGTGCGGAAGTGAAATTTGAAGGCGATACCCTGGT<br>AAACCGCATTGAGCTGAAAGGCATTGACTTTAAAGAAGACGGCAATATCCTGGGCCATAAGCTGGAATACAATTTTAACAGCCACAATGTT<br>TACATCACCGCCGATAAACAAAAAAATGGCATTAAAGCGAATTTTAAAATTCGCCACAACGTGGAGGATGGCAGCGTGCAGCTGGCTGATC<br>ACTACCAGCAAAACACTCCAATCGGTGATGGTCCTGTTCTGCTGCCAGACAATCACTATCTGAGCACGCAAAGCGTTCTGTCTAAAGATCC<br>GAACGAGAAACGCGATCACATGGTTCTGCTGGAGTTCGTAACCGCAGCGGGCATCACGCATGGTATGGATGAACTGTACAAAGAATTCGC<br>TGAAGCTGCTGCTAAAGAAGCTGCTGCTAAAGAAGCTGCTGCTAAAGAAGCTGCTGCTAAAGCTACCACCAAATCTGCTTTCACCTGCACC<br>GCTACCACCGCTTCTAACTACGCTCACGTTCAGGCTGGTCGTGCTCACGACTCTGGTGGTATCGCTTACGCTAACGGTTCTAACCAGTCTA<br>TGGGTCTGGACAACCTGTTCTACACCTCTACCCTGGCTCAGACCGCTGCTGGTTACTACATCGTTGGTAACTGCCCGCTCGAG

Legend: The sfGFP sequence is highlighted in green, while the SBD domain sequence is highlighted in orange and the linkers (flexible or rigid) are highlighted in purple. The underlined sequences represent the restriction sites for enzymes NdeI (CATATG), EcoRI (GAATTC) or XhoI (CTCGAG). The C-terminal 6xHis tag and stop codon of all proteins are not shown, as they are encoded natively by the pET28a(+) and pET29b(+) plasmids.

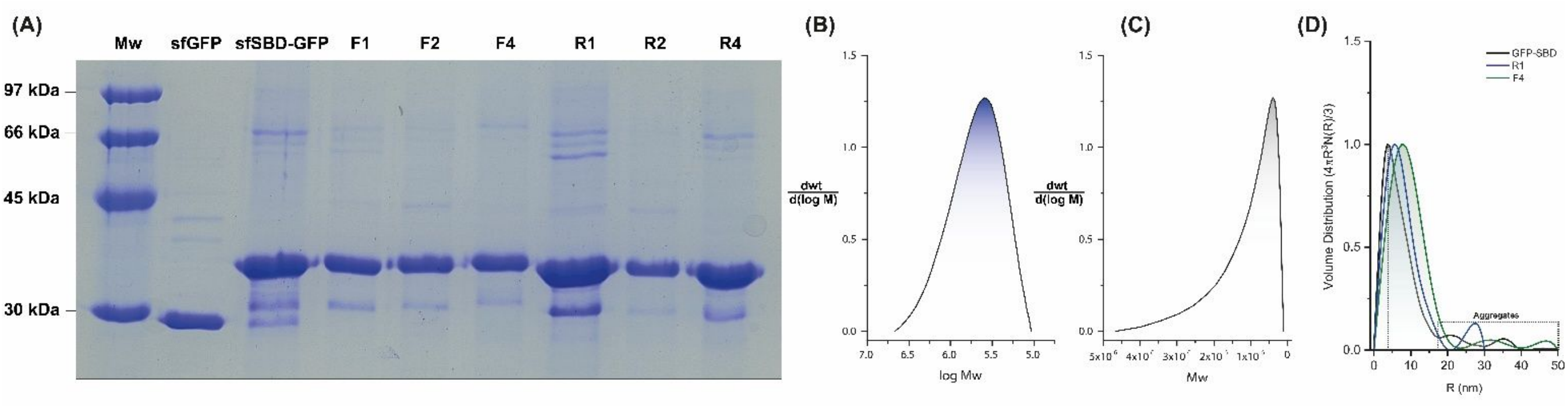

Figure S1. SDS-PAGE of all fusion proteins (A), SEC Mw profile of refractive index derived differential weight fraction of PHB performed in $CHCl_3$ (B and C). SAXS volume distribution profile of sfGFP-SBD, R4 and F4 (D).

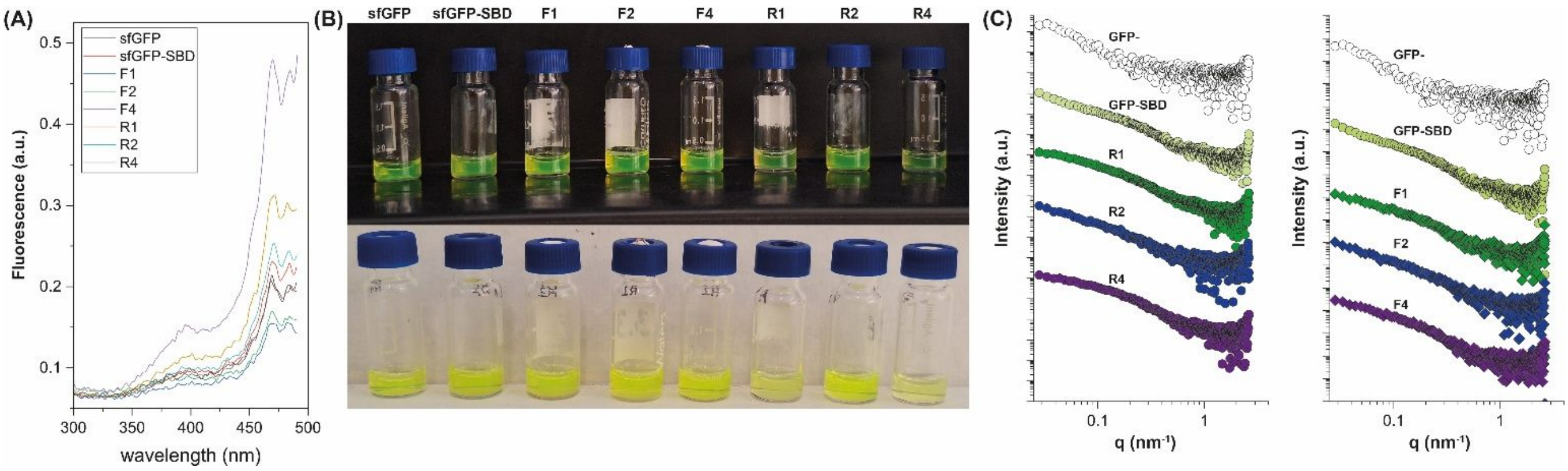

Figure S2. Emission fluorescence spectra (A) of all purified proteins with respect sample image (B). SAXS profiles of all samples in TrisHCl buffer pH 8 (C).

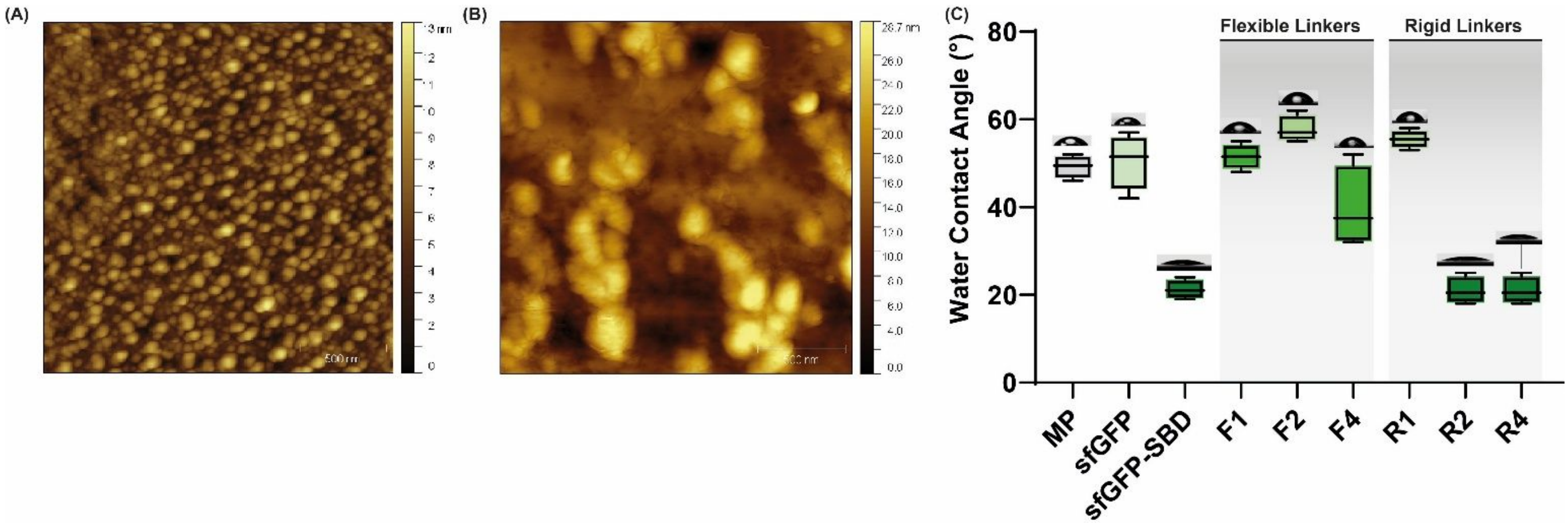

Figure S3. AFM imagens of QCM-D sensor after cleaning (A) and after MP deposition (B). Water contact angle performed over QCM-D sensors after protein deposition and rinsing (C).

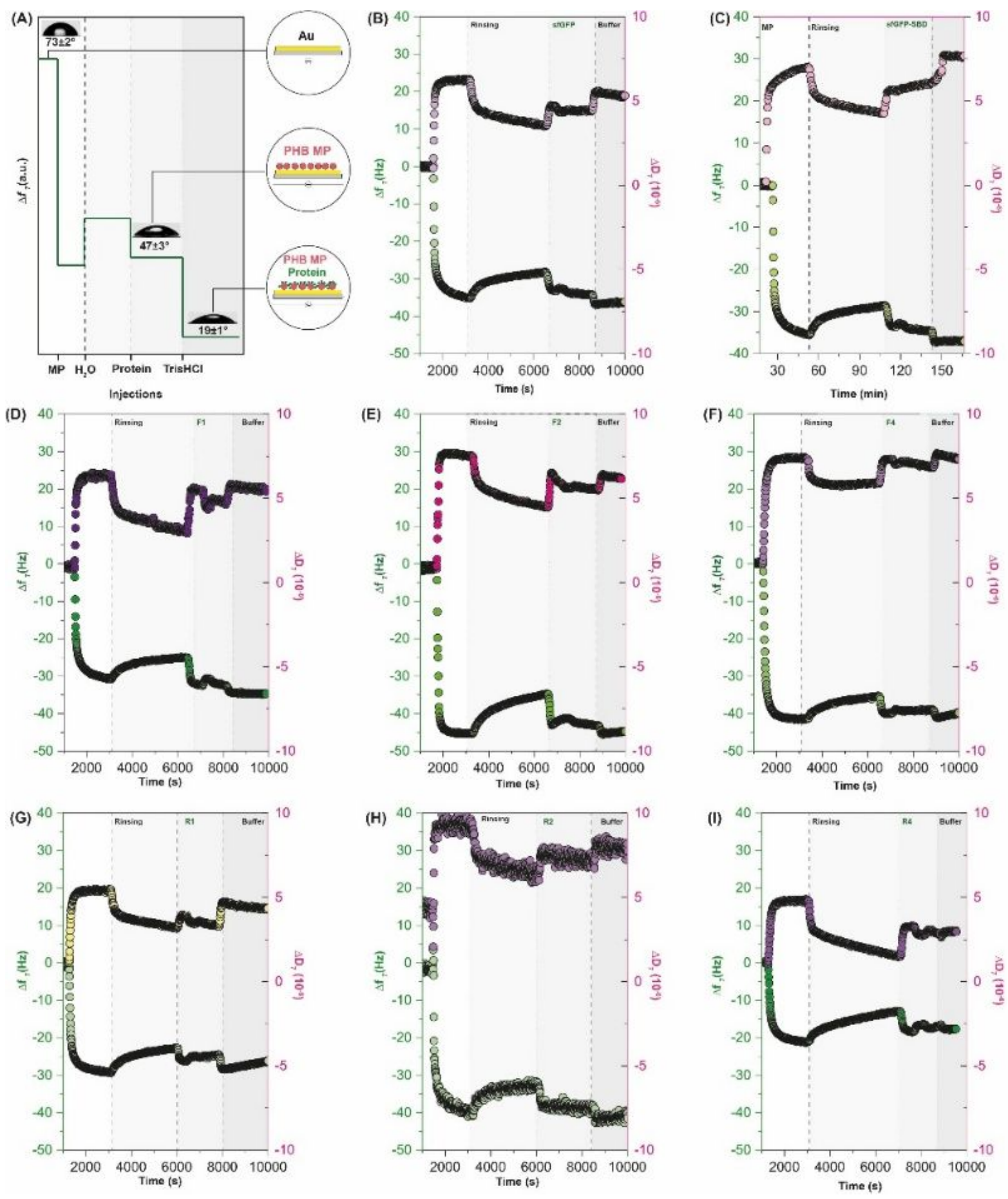

Figure S4. Schematic representation of QCM-D experiments and surface characterization by contact angle (A). Adsorption profile (7th harmonic) of MP and sfGFP (B), sfGFP-SBD (C), F1 (D), F2 (E), F4 (F), R1 (G), R2 (H) and R4 (I).

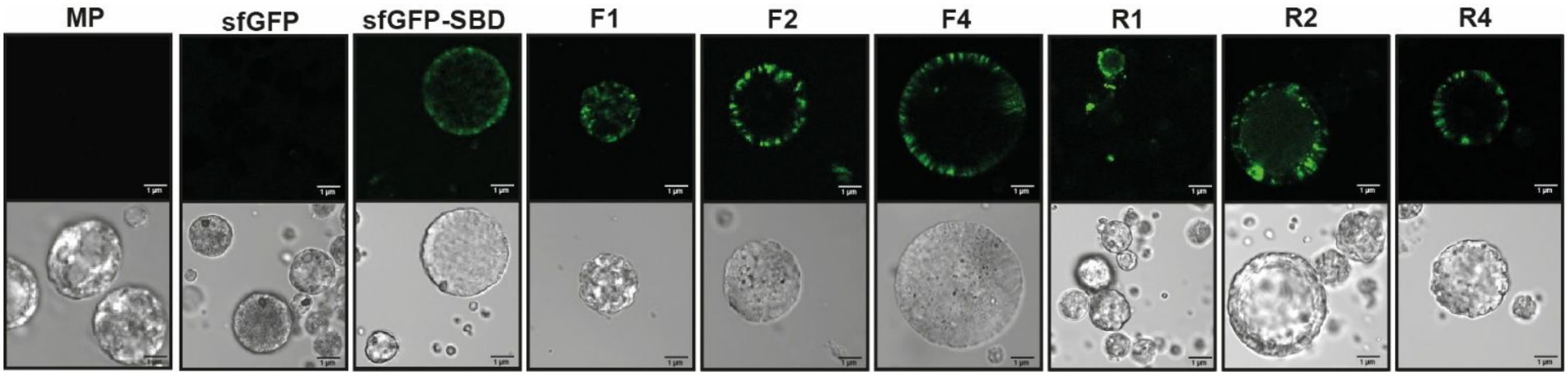

Figure S5. CLSM images of MP after protein immobilization for fluorescent (top) and differential interference contrast (bottom).

Table S2. Hydrodynamic diameter, polydispersity and ζ-potential of NP incubated with the proteins after dialysis.

Sample	Diameter (nm)	PDI	ζ-Potential (mV)
NP	190±75	0.25±0.006	-10.1±0.1
NP:sfGFP	190±70	0.37±0.01	-11.4±0.1
NP:sfGFP-SBD	165±82	0.26±0.02	-13.4±0.5
NP:F1	164±93	0.22±0.02	-12.1±0.3
NP:F2	165±80	0.15±0.02	-12.3±0.1
NP:F4	165±90	0.15±0.06	-11.4±0.2
NP:R1	190±78	0.24±0.05	-10.1±0.2
NP:R2	165±85	0.22±0.02	-16.6±0.3
NP:R4	190±75	0.25±0.01	-12.9±0.4

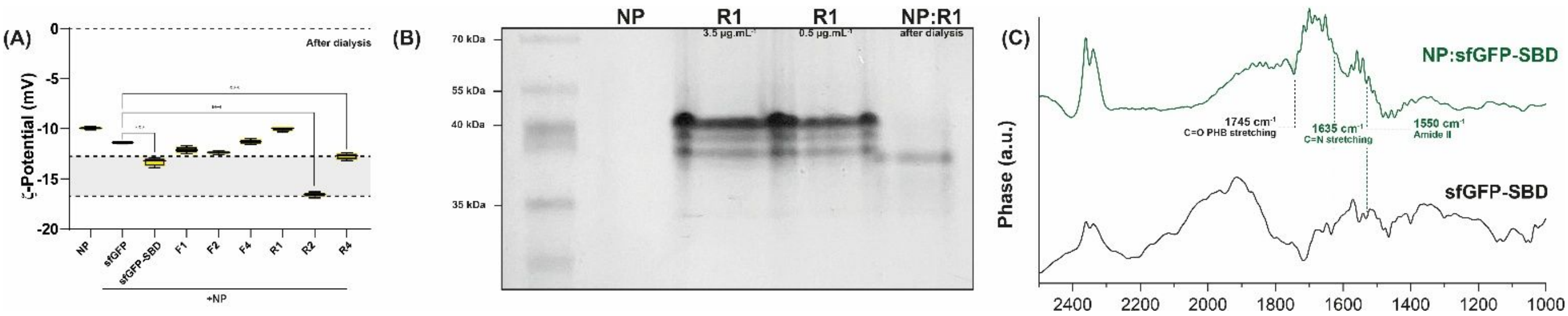

Figure S6. ζ-potential of NP after protein incubation and dialysis (A) and native polyacrylamide gel electrophoresis of NP, R1, and the NP:R1 after dialysis (B). s-SNOM IR spectra of NP:sfGFP-SBD and sfGFP-SBD (C).

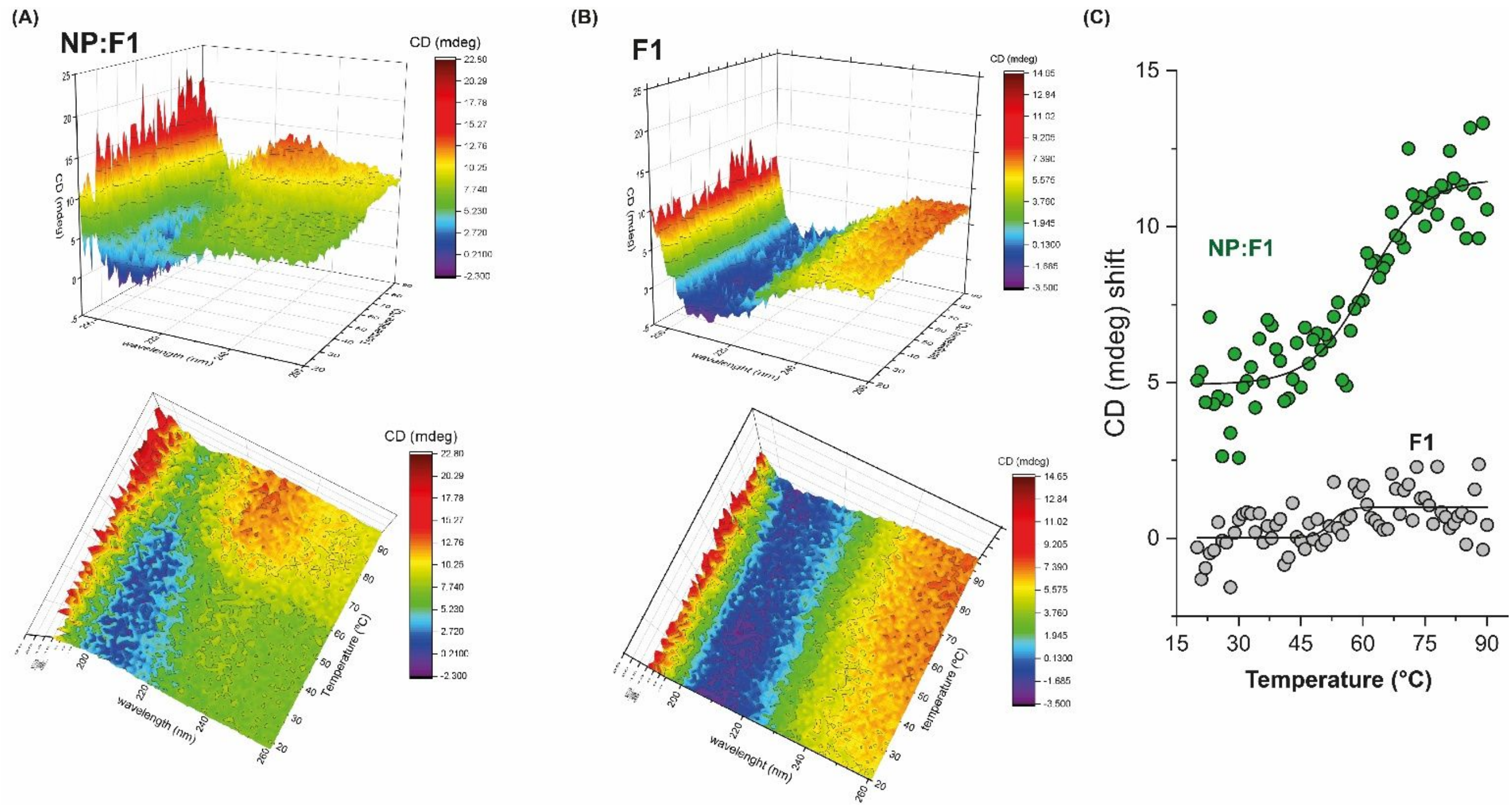

Figure S7. Ramp-temperature Circular dichroism analysis of spectra of NP:F1 (A) and F1 (B) from 20 to 90°C. Bolstzmann-fitted CD shift of both samples for Tm determination (C).
